# The Role of Noninvasive Endpoints in Predicting Long-Term Outcomes in Pulmonary Arterial Hypertension

**DOI:** 10.1007/s00408-019-00289-2

**Published:** 2019-11-13

**Authors:** Samantha L. Wronski, Margaret Mordin, Kim Kelley, Rebekah H. Anguiano, Peter Classi, Eric Shen, Scott Manaker

**Affiliations:** 1 RTI Health Solutions, Research Triangle Park, NC USA; 2 RTI Health Solutions, Ann Arbor, MI USA; 3 Rx Trusted Advisors, Phoenix, AZ USA; 4 Department of Pharmacy Practice, University of Illinois at Chicago, Chicago, IL USA; 5 United Therapeutics, Research Triangle Park, NC USA; 6 Department of Medicine, Perelman School of Medicine at the University of Pennsylvania, Philadelphia, PA USA

**Keywords:** PAH, Noninvasive endpoint, Risk assessment

## Abstract

**Background:**

Until recently, many clinical trials in patients with pulmonary arterial hypertension (PAH) evaluated exercise capacity with 6-minute walk distance (6MWD) as the primary endpoint. Common secondary endpoints include PAH functional class (FC), which assesses symptoms, and either brain natriuretic peptide (BNP) or the inactive N-terminal cleavage product of its prohormone (NT-proBNP), which assesses cardiac function.

**Objective:**

Examine the relationships among 6MWD, FC, and BNP/NT-proBNP measured at baseline or follow-up with long-term outcomes in PAH studies.

**Methods:**

Relevant literature from January 1990 to April 2018 were obtained by searching PubMed, Embase, and Cochrane. Articles in English reporting on associations between 6MWD, FC, or BNP/NT-proBNP and outcomes in PAH were identified. Each endpoint was evaluated individually. Prespecified inclusion and exclusion criteria were applied at level 1 (titles/abstracts) and level 2 (full-text review).

**Results:**

The database search yielded 836 unique records; 65 full-text articles were reviewed. Twenty-five studies were eligible for inclusion. Findings supported the importance of measuring PAH noninvasive endpoints in predicting long-term outcomes. Patients with shorter or decreased 6MWD, poor (III/IV) or declining FC (e.g., from II to III), or elevated or increasing BNP/NT-proBNP had a higher risk of death and costly events (e.g., hospitalization, lung transplant). FC also predicted health care resource utilization and costs. Collectively, these endpoints establish risk groups that predict likelihood of complications from PAH or death.

**Conclusion:**

Assessment of 6MWD, FC, and BNP/NT-proBNP provides low-cost, efficient, and noninvasive means of predicting long-term health and economic outcomes in patients with PAH.

## Introduction

With an estimated prevalence of 10.6–12.4 cases per million [1], pulmonary arterial hypertension (PAH) is a rare chronic and progressive disease characterized by increased pulmonary vascular resistance that can result in death due to right heart failure [2]. Numerous available treatments for PAH [3] have been evaluated in clinical trials using a variety of endpoints [4–6]. In the past two decades, PAH study design and duration shifted from short-term trials assessing noninvasive endpoints to long-term event-driven trials [7, 8].

Six-minute walk distance (6MWD), functional class (FC), and indicators of right ventricular function (i.e., brain natriuretic peptide [BNP]/the active N-terminal cleavage product of its prohormone [NT-proBNP], described in Table 1) are among the commonly used short-term primary and secondary noninvasive endpoints in clinical PAH trials [9, 10]. In a literature review examining 126 pulmonary hypertension (PH) clinical trials (78% in PAH) from 1985 to 2013, surrogate measures were primary endpoints in 95% of trials and secondary endpoints in 33% of trials [9]. 6MWD and FC were among the noninvasive endpoints that were used significantly more frequently (*P* < 0.0001) [9]. The latest 2015 European Society of Cardiology/European Respiratory Society treatment guidelines [11] and registry studies such as COMPERA [12] and REVEAL [13] also include 6MWD, FC, and BNP/NT-proBNP as important components of risk assessment. Collectively, 6MWD, FC, and BNP/NT-proBNP serve as measurable prognostic indicators of the distal outcomes of morbidity and mortality that may be assessed early in order to determine treatment course and improve outcomes [14, 15]. However, the clinical relevance and ability of these noninvasive endpoints to consistently correlate with key indicators of disease progression, such as hospitalization and death, has received mixed support [16–25]. This, coupled with improvements in survival and quality of life that have allowed recent clinical trials to follow patients with PAH for 4–6 years [7], has led to the prominence of mortality and morbidity as endpoints.

**Table 1:** Description of noninvasive endpoints

Noninvasive endpoint	Description
6MWD	6MWD assesses disease severity by measuring the distance an individual is able to walk over 6 min on a hard, flat surface [16]
WHO FC NYHA FC	Level of FC, determined according to WHO FC or NYHA FC, ranges from I–IV and is physician assessed [16]. WHO FC was adopted in 1998 as a modified version of NYHA FC, which was developed in 1928 [62]. WHO FC classes are defined as follows [62]: I: Patients with PH in whom there is no limitation of usual physical activity; ordinary physical activity does not cause increased dyspnea, fatigue, chest pain, or presyncope II: Patients with PH who have mild limitation of physical activity. There is no discomfort at rest, but normal physical activity causes increased dyspnea, fatigue, chest pain, or presyncope III: Patients with PH who have a marked limitation of physical activity. There is no discomfort at rest, but less than ordinary activity causes increased dyspnea, fatigue, chest pain, or presyncope IV: Patients with PH who are unable to perform any physical activity at rest and who may have signs of right ventricular failure. Dyspnea and/or fatigue may be present at rest, and symptoms are increased by almost any physical activity
BNP NT-proBNP	BNP is a neurohormone released by the myocardium, predominantly in the ventricles secreted in response to changes in pressure inside the heart as measured through a blood test. Studies may measure BNP directly or NT-proBNP, which is the nonactive prohormone released from the same molecule that produces BNP [63]

*6MWD* 6-minute walk distance, *BNP* brain natriuretic peptide, *FC* functional class, *NT-proBNP* N-terminal-prohormone BNP, *NYHA FC* New York Heart Association Functional Class, *PH* pulmonary hypertension, *WHO FC* World Health Organization Functional Class

Morbidity and mortality, a term reflecting clinical worsening and disease progression [16, 23, 26], provide a robust demonstration of efficacy, safety, and long-term benefits of treatments for PAH [23]. The use of clinical worsening or disease progression as a primary endpoint in phase 3 trials was endorsed by the Task Force on End Points and Clinical Trial Design of both the Fourth and Fifth World Symposium on Pulmonary Hypertension and the 2008 Dana Point Task Force on End Points and Clinical Trial Design [16]. However, comparison of treatment efficacy across trials may be hindered by the varying definitions used for clinical worsening, as seen when comparing the definitions used in the AMBITION [27], GRIPHON [28], SERAPHIN [29], and FREEDOM EV [30–32] clinical trials. While it has been argued that the composite endpoint of clinical worsening is more clinically meaningful than noninvasive endpoints [19, 25], all four trials include 6MWD and FC in their definitions of clinical worsening, despite other differences. Additional components in the definition of clinical worsening, such as death, hospitalization, and lung transplant, require long-term follow-up to assess, and despite clearly indicating clear and undisputable indicators of ultimate treatment efficacy and safety, they cannot be used to assess clinical risk in the day-to-day care of patients with PAH that guide treatment decisions.

There is a need to revisit the optimal duration of future trials [7] and include clinically meaningful endpoints that reflect how patients feel and function [33]. In addition, the use of universal endpoints in PAH clinical trials and observational studies would better inform health care providers, decision makers, and payers on the value of targeted pharmacotherapies and combination therapies for patients with PAH [16]. 6MWD, FC, and BNP/NT-proBNP are universal endpoints, routinely used in clinical risk assessment, that can be assessed short-term (12–16 weeks [23]). The present review examines the value of 6MWD, FC, and BNP/NT-proBNP by summarizing the literature supporting the relationship between these noninvasive endpoints and long-term clinical and economic outcomes.

## Methods

A literature review was conducted on April 13, 2018, in PubMed, Embase, and the Cochrane Database of Systematic Reviews using a search strategy (Table 2) that included Medical Subject Headings (MeSH) and key words for disease (e.g., pulmonary arterial hypertension), endpoints (e.g., 6MWD, FC, BNP), clinical importance (e.g., survival, mortality), and economic importance (e.g., costs, readmission, economics). Inclusion criteria incorporated studies published after January 1, 1997, in English and human subjects; comments, letters, or editorials were excluded. Bibliographies of relevant review articles were reviewed for any pertinent articles unidentified in the original search. To obtain information from relevant unpublished studies, a search of 2016–2017 conference abstracts via Embase was performed, including the American Thoracic Society International Conference, American College of Chest Physicians (CHEST) Annual Meeting, CHEST World Congress Annual Meeting, and the International Society for Pharmacoeconomics and Outcomes Research International Meeting. Titles and abstracts of records were reviewed (level 1 screening) according to the objectives and inclusion and exclusion criteria. Included studies, defined using PICOS (population, intervention, comparison, outcome, study type), had the following:

- A primarily adult population (≥ 18 years) with PAH from a WHO group 1 etiology
- Any intervention or comparator
- At least one of the noninvasive endpoints—6MWD, FC, BNP/NT-proBNP (Table 1)—and reported on the relationship between a noninvasive endpoint and a clinical or economic outcome of interest (note that the literature was searched for individual associations between each of the endpoints and PAH outcomes)
- An interventional (e.g., randomized controlled trials) or noninterventional (e.g., observational, prospective, retrospective, database, and/or registry studies) design, with ≥ 75 patients, or a relevant literature review.

**Table 2:** PubMed search strategy (search conducted April 13, 2018)

Search number	Search terms	Number of results
Disease		
1	“Familial Primary Pulmonary Hypertension”[Majr] OR “pulmonary arterial hypertension”[Title/Abstract] OR “primary pulmonary hypertension”[Title/Abstract] OR “idiopathic pulmonary hypertension”[Title/Abstract]	9301
Endpoints		
2	1 AND (“six minute walk”[Title/Abstract] OR “6 min walk”[Title/Abstract] OR “6MWD”[Title/Abstract] OR “6MWT”[Title/Abstract] OR “New York Heart Association Functional Class”[Title/Abstract] OR “NYHA functional class”[Title/Abstract] OR “NYHA FC”[Title/Abstract] OR “World Health Organization Functional Class”[Title/Abstract] OR “WHO functional class”[Title/Abstract] OR “WHO FC”[Title/Abstract] OR “brain natriuretic peptide”[Title/Abstract] OR “pro-brain natriuretic peptide”[Title/Abstract] OR “BNP”[Title/Abstract] OR “NT-proBNP”[Title/Abstract] OR “Natriuretic Peptide, Brain”[Majr])	1208
Clinical importance		
3	2 AND (“Disease Progression”[Majr] OR “Familial Primary Pulmonary Hypertension/mortality”[Majr] OR “Mortality”[Majr] OR “Familial Primary Pulmonary Hypertension/complications”[Majr] OR “Survival”[Majr] OR “Comorbidity”[Majr] OR “Quality of Life”[Majr] OR “quality of life”[Title] OR risk*[Title] OR surviv*[Title] OR mortalit*[Title] OR death*[Title] OR prognos*[Title] OR “disease progression”[Title/Abstract] OR “disease exacerbation”[Title/Abstract] OR complicat*[Title/Abstract] OR sequelae[Title/Abstract] OR comorbidit*[Title/Abstract] OR multimorbidit*[Title/Abstract] OR fatal*[Title/Abstract] OR “life quality”[Title/Abstract] OR “QoL”[Title/Abstract] OR “hrqol”[Title/Abstract] OR “hrql”[Title/Abstract])	370
Economic importance		
4	2 AND (“Patient Readmission”[Majr] OR “Hospitalization”[Majr] OR “Length of Stay”[Majr] OR “Fees and Charges”[Majr] OR “Health Care Costs”[Majr] OR “Costs and Cost Analysis”[Majr] OR “Economics”[Majr] OR “Economics, Hospital”[Majr] OR “Economics, Medical”[Majr] OR “Economics, Nursing”[Majr] OR “Economics, Pharmaceutical”[Majr] OR “Budgets”[Majr] OR “Health Expenditures”[Majr] OR “Cost of Illness”[Majr] OR “Cost–Benefit Analysis”[Majr] OR hospital*[Title/Abstract] OR “length of stay”[Title/Abstract] OR “stay length”[Title/Abstract] OR readmission[Title/Abstract] OR readmit*[Title/Abstract] OR cost[Title/Abstract] OR costs[Title/Abstract] OR costly[Title/Abstract] OR economic*[Title/Abstract] OR fiscal[Title/Abstract] OR fee[Title/Abstract] OR fees[Title/Abstract] OR expenditure*[Title/Abstract] OR budget*[Title/Abstract])	172
Exclusions		
5	“Animals”[MeSH] NOT “Humans”[MeSH]	2,060,466
6	“Comment”[Publication Type] OR “Letter”[Publication Type] OR “Editorial”[Publication Type]	1,104,425
7	(“Child”[MeSH] OR “Infant”[MeSH] OR “Adolescent”[MeSH] OR child*[Title/Abstract] OR infant*[Title/Abstract] OR newborn*[Title/Abstract] OR adolescen*[Title/Abstract]) NOT (“Adult”[MeSH] OR adult*[Title/Abstract] OR elder*[Title/Abstract] OR senior citizen*[Title/Abstract] OR middle age*[Title/Abstract])	937,574
Total		
8	(#3 OR #4) NOT (#5 OR #6 OR #7)	460

Search terms and limits were adapted for searching in Embase and the Cochrane Database of Systematic Reviews. Limits include 1997–present; English; humans; adults; no comments, letters, editorials

Full texts of included studies were reviewed (level 2 screening) using the same relevance criteria applied at level 1. That is, full-text articles were reviewed in detail, and the inclusion and exclusion criteria applied at level 1 (title/abstract screening) were applied to evaluate the appropriateness for inclusion.

## Results

### Literature Review

**Fig. 1:**
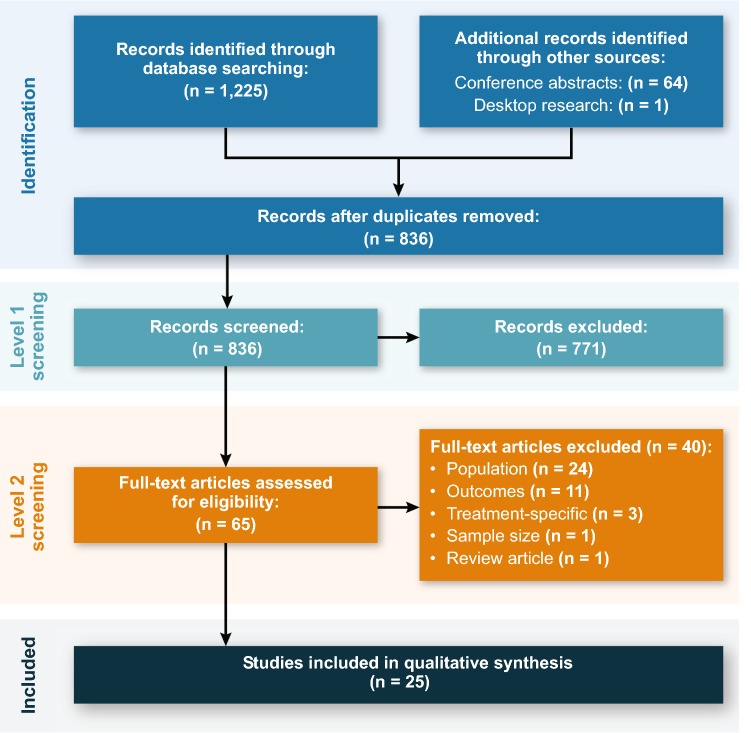
Literature review flow diagram

Figure 1 summarizes the literature review, which identified 836 unique studies for level 1 screening, including 64 conference abstracts and 1 study identified through desktop research. Of these, 65 records were selected for level 2 screening; no conference abstracts were deemed eligible for inclusion. Twenty-five studies, summarized in Table 3, were selected for inclusion according to predefined inclusion/exclusion criteria. Although outcomes, such as risk of death or the combined endpoint of risk of death or lung transplant, were consistently defined across the literature, clinical worsening was defined differently in each of the three studies evaluating it as an outcome [2, 34, 35]; definitions used are noted in Table 3.

**Table 3:** Description of literature evaluating relationships between noninvasive endpoints and health outcomes

References	Study type	Description	Timeframe	Sample size	Population
Frantz et al. [52]	Prospective registry (REVEAL)	REVEAL Registry	2006–2012	1426	WHO group 1 PAH (confirmed by right-sided heart catheterization)
Kylhammar et al. [55]	Observational registry (SPAHR)	Study of incident cases of patients classified into PAH risk groups, determined on the basis of multiple noninvasive measurements, including WHO FC, 6MWD, NT-proBNP, and echocardiography imaging and hemodynamics	January 1, 2008–March 1, 2016	530	PAH diagnoses included idiopathic/familial PAH, CTD-associated PAH, CHD-associated PAH, or other forms of associated PAH (drug- and toxin-induced, HIV-associated, and portal-hypertension-associated PAH)
Snipelisky et al. [47]	Retrospective	Review of patients at University of Pittsburgh Medical Center. NYHA FC was extracted from electronic medical record. From a sample of 273 patients, 163 had documented serum albumin concentration and comprised the final study population	March 2001–August 2008	163	WHO group 1 PAH
Souza et al. [37]	RCT	SERAPHIN multicenter, randomized controlled, event-driven study assessing the long-term efficacy and safety of macitentan	May 2008–April 2012	742	PAH (WHO FC II-IV) diagnosed by right heart catherization with idiopathic PAH, heritable PAH, or PAH related to connective tissue disease, repaired congenital systemic-to-pulmonary shunts, HIV infection, drug use, or toxin exposure
Weatherald et al. [45]	Retrospective registry (French Pulmonary Arterial Hypertension Network Registry)	Review of patients enrolled in a registry	2006–2016	981	Diagnosed with idiopathic, heritable, or drug-induced PAH who had at least 1 follow-up RHC
Zelniker et al. [38]	Prospective registry (COMPERA)	Multinational, prospective registry that enrolls patients with newly diagnosed PAH who receive targeted medical therapy; all patients underwent right heart catherization	June 2007–January 2016	2391; Survival analysis = 2178	Newly diagnosed PAH; etiologies included idiopathic/drug-associated or hereditary PAH, connective tissue disease, HIV-associated PAH, portopulmonary hypertension, and congenital heart disease
Boucly et al. [54]	Retrospective registry	Review of all incident (newly diagnosed) patients enrolled in a French registry	2006–2016	603	Idiopathic, heritable, or drug- and toxin-induced PAH
Dufour et al. [6]	Retrospective	Observational cohort study based on de-identified administrative claims data from the Humana Research Database. Data sources included medical and pharmacy claims, and enrollment records. ~ 70% of the database included patients with Medicare Advantage plans, and ~ 30% included patients with commercial insurance	January 1, 2009–June 30, 2014	476	Patients had at least one claim for a PAH-specific medication during the study period, and at least one medical claim with one relevant diagnosis code associated with PH in any position on the administrative medical claim form or at least one medical claim with a CPT or ICD-9-CM code indication at right heart catherization during the identification period; ICD-9 diagnosis codes included 416.0 (primary pulmonary hypertension), 416.8 (other chronic pulmonary heart diseases), or 416.9 (chronic pulmonary heart disease, unspecified)
Hoeper et al. [12]	Prospective registry (COMPERA)	Patients with newly diagnosed PAH were classified according to risk using the strategy proposed by the European PH guidelines, which consider WHO FC, 6MWT, BNP/NT-proBNP, right atrial pressure, cardiac index, and mixed venous oxygen saturation	January 1, 2009–December 1, 2016	1588	Treatment-naive, newly diagnosed PAH; etiologies included idiopathic/drug-associated or hereditary PAH, connective tissue disease, HIV-associated PAH, portopulmonary hypertension, and congenital heart disease
Tang et al. [35]	Prospective	Analysis of patients who were admitted to Fuwai Hospital and underwent symptom-limited cardiopulmonary exercise testing Clinical worsening was defined as the time from cardiopulmonary exercise testing to the first event, which included the following: all-cause mortality, lung transplant, hospitalization for worsening of PAH, the need for epoprostenol therapy, and interventional procedures (performance of balloon atrial septostomy)	November 11, 2010–June 25, 2015	210	Newly diagnosed idiopathic PAH
Ghofrani et al. [2]	RCT	Patients from the PATENT-1 study who entered the PATENT-2 open-label extension Clinical worsening was defined as the first occurrence of any of the following events: death, heart or lung transplant, atrial septostomy, admission to hospital due to worsening of pulmonary arterial hypertension, start of new specific pulmonary arterial hypertension treatment or modification of existing prostanoid treatment (increase in dosage or frequency of existing prostanoid therapy, or initiation of an intravenous prostanoid), persistent decrease of greater than 15% from baseline in 6MWD, and persistent worsening of WHO FC	March 12, 2009–March 1, 2014	396	PAH etiologies included idiopathic PAH, familial PAH, connective tissue disease, systemic-sclerosis–associated PAH, congenital heart disease, portal pulmonary, anorexigen or amphetamine-associated PAH
Huang et al. [34]	Retrospective	Analysis of patients from the Southwest Ontario Pulmonary Hypertension Clinical of the Western University Clinical worsening was defined as either: development of right heart failure, hospital admission for PAH, referral for lung transplant or initiation of prostanoids after oral therapy failed	Not specified	100	WHO group I PAH; diagnoses included idiopathic PAH, CTD PAH, and CHD PAH
Ozpelit et al. [36]	Prospective	Consecutive adult patients with definitive PAH who attended the PAH Clinic, Department of Cardiology, School of Medicine, Dokuz Eylul University, Izmir, Turkey	January 2008–June 2014	101	Definitive PAH; patients with overt infections disease at the time of PAH diagnosis were excluded
Zelniker et al. [43]	Prospective	Patients enrolled in the outpatient department of the University Hospital of Heidelberg, Germany (referral center for PAH patients)	January 2010–May 2010	95	Confirmed PAH (Dana point group 1); diagnoses were categorized as idiopathic PAH, PAH and connective tissue disease, other
Ehlken et al. [53]	Prospective	German prospective analysis, patients with severe PAH receiving exercise training plus medical therapy compared with patients who received medical therapy alone	Semistructured phone interviews were performed in April 2007 to assess survival and clinical status of the patients	Training group = 58 Retrospective control group = 46	PAH etiologies included idiopathic and familial; PAH associated with collagen vascular disease, congenital systemic-to-pulmonary shunts, portal hypertension, or HIV; PH associated with the following lung diseases: chronic obstructive pulmonary disease, interstitial lung disease, chronic thromboembolic pulmonary hypertension, or other causes
Barst et al. [49]	Prospective Registry (REVEAL)	Using the REVEAL registry, patients with were classified as improved, unchanged, or worsened according to their change in FC from enrollment to first follow-up within 1 year (mean ± SD: 4 ± 3 months)	Not specified (REVEAL data spanned 2006–2012)	982	WHO group I NYHA/WHO FC III PAH
Fritz et al. [40]	Retrospective (analysis of 2 RCTs)	Pooled analysis of patients enrolled in 2 RCTs (Ambrisentan in Pulmonary Arterial Hypertension, Randomized, Double-Blind, Placebo-Controlled, Multicenter, Efficacy Study 1/2 [ARIES-1 and ARIES-2]) who had 2-year follow-up	January 2004–February 2006	370	PAH etiologies were idiopathic, connective tissue disease, and other (unspecified)
Tiede et al. [50]	Prospective registry (Giessen Pulmonary Hypertension Registry Study)	Registry containing a total of ~ 2500 patients at a single specialized referral center (the Giessen Pulmonary Hypertension Center, Universities of Giessen and Marburg Lung Center, University Hospital Giessen, Giessen, Germany)	1991–2013	~ 700	Newly diagnosed WHO group 1 PAH (according to Dana Point classification)
Batal et al. [41]	Retrospective	Review of records of consecutive patients with PAH who underwent diagnostic RHC at the Cleveland Clinic, consisting of those who died within 2 years (reduced survival) and those who survived ≥ 5 years (long survival group)	February,1996–January, 2006	Reduced survival = 21; long survival = 60	PAH etiologies were idiopathic and scleroderma
Nickel et al. [46]	Prospective	German cohort database study from Hanover Medical School of patients who had undergone at least 1 follow-up RHC within the first year after PAH-targeted therapy had been initiated	1999–2009	109	Newly diagnosed with idiopathic PAH
Benza et al. [44]	Retrospective (analysis of three RCTs)	Review of patients who were enrolled in three trials (P01: 04, 05, 06) treated with subcutaneous treprostinil	June 25, 1998–December 1, 2003	811	PAH etiology: idiopathic, associated PAH, connective tissue disease, congenital heart disease, portopulmonary hypertension
Kane et al. [48]	Retrospective	Retrospective single-center study of consecutive patients at the Mayo Clinic Rochester	January 1, 1995–December 31, 2004	484	Fulfilled the contemporary diagnostic criteria for WHO group 1 PAH. Diagnoses included idiopathic, familial, or anorexigenic PAH; PAH in the setting of connective tissue disease; and PAH associated with congenital systemic-to-pulmonary shunts, portal hypertension, and HIV
Mauritz et al. [51]	Retrospective	Analysis of patients from the Department of Pulmonology of VU Medical Center of Amsterdam (The Netherlands)	November 2002–September 2009	198	WHO group 1 PAH diagnoses included idiopathic PAH, associated connective tissue disease, associated portal hypertension, associated HIV infection, drug- and toxin-induced PAH, other
Benza et al. [39]	Prospective registry (REVEAL)	Patients consecutively enrolled in the US REVEAL registry	Not specified (registry began in 2006)	2716	WHO group 1 PAH including idiopathic and familial PAH
Humbert et al. [42]	Prospective registry (French Network on Pulmonary Hypertension Prospective Registry)	Consecutive patients seen in 17 university pulmonary vascular centers	October 2002–October 2003 and followed for 3 years	354 (56 were incident and 298 were prevalent cases)	Idiopathic, familial, or anorexigen-associated PAH

*6MWD* 6-minute walk distance, *6MWT* 6-Minute Walk Test, *BNP* brain natriuretic peptide, *CHD* congenital heart disease, *CPT* Current Procedural Terminology, *CTD* connective tissue disease, *FC* functional class, *ICD-9-CM International Classification of Diseases, Ninth Revision, Clinical Modification*, *NT-proBNP* BNP/the inactive N-terminal cleavage product of its prohormone, *NYHA* New York Heart Association, *PAH* pulmonary arterial hypertension, *PH* pulmonary hypertension, *RCT* randomized controlled trial, *RHC* right heart catheterization, *SD* standard deviation, *US* United States, *WHO* World Health Organization

### 6MWD

Thirteen studies evaluated the relationship between 6MWD and death (*n* = 10), death or lung transplant (*n* = 3), PAH-related death or hospitalization (*n* = 1), and risk of experiencing a costly event indicative of clinical worsening (*n* = 2) (Table 4).

**Table 4:** Summary of relationships between noninvasive endpoints (6MWD, FC, BNP/NT-proBNP, and risk groups) and outcomes in the literature

References	Noninvasive endpoint	Outcome	Observed relationship*
6MWD			
Souza et al. [37]	6MWD	PAH-related death or hospitalization over a maximum of 36 months from follow-up (median treatment duration: 2.2 years)	Patients with 6MWD in lower quartiles at baseline (reference: Q1 ≤ 300 m) or 6-month follow-up (reference: Q1 ≤ 348 m) had an increased risk of PAH-related death or hospitalization Patients with 6MWD below the median (≤400 m) at 6-month follow-up had an increased risk of PAH-related death or hospitalization Patients with a 6MWD ≤ 400 m at 6 months had a similarly poor long-term outcome regardless of whether their baseline 6MWD was > 400 m or ≤ 400 m
	6MWD	All-cause death over a maximum of 36 months from follow-up (median treatment duration: 2.2 years)	Patients with 6MWD in lower quartiles at baseline (reference: Q1 ≤ 300 m) or 6-month follow-up (reference: Q1 ≤ 348 m) had an increased risk of all-cause death Patients with a 6MWD below the median (≤400 m) at 6-month follow-up had an increased risk of all-cause death Patients with a 6MWD ≤ 400 m at 6 months had a similarly poor long-term outcome regardless of whether their baseline 6MWD was > 400 m or ≤ 400 m
Weatherald et al. [45]	6MWD	Death or lung transplant	Patients with shorter baseline 6MWD (per 10 m) had a greater risk of death or lung transplant over a median follow-up of 2.8 years (IQR: 1.1–4.6) in univariable and multivariable analyses At first follow-up (median time to first follow-up right heart catherization was 4.6 months [IQR: 3.7–7.8]), patients with shorter 6MWD (per 10 m) had an increased risk of death or lung transplant over a median follow-up of 2.8 years (IQR: 1.1–4.6) in univariable and multivariable analyses
Zelniker et al. [38]	6MWD	Death at 1 year	Patients with shorter 6MWD or 6MWD below a cutoff of < 165 m at baseline have an increased risk of death at 1 year; similar findings at follow-up^a^ Patients with decreasing 6MWD between baseline and follow-up^a^ had an increased risk of death at 1 year
Ghofrani et al. [2]	6MWD	Death	Patients with shorter 6MWD or 6MWD less than the median (< 380 m) at baseline had a significantly increased risk of death in bivariate Cox proportional hazards models; similar findings for shorter 6MWD or 6MWD less than the median (< 418 m) at follow-up in univariate analysis
	6MWD	Clinical worsening (see Table 3 for definition)	Patients with shorter 6MWD at baseline or declining 6MWD between baseline and follow-up had significantly increased risk of clinical worsening in bivariate Cox proportional hazards models; similar findings for shorter and declining 6MWD at follow-up in univariate analysis
Huang et al. [34]	6MWD	Death or lung transplant	Patients with shorter 6MWD or 6MWD ≤ 342 m at baseline had an increased risk of death or lung transplant; similar findings for declines in 6MWD ≥ 35 m or ≥ 8% at 6-month follow-up
	6MWD	Clinical worsening (see Table 3 for definition)	Patients with shorter 6MWD or 6MWD ≤ 342 m at baseline had an increased risk of clinical worsening; similar findings for declines in 6MWD ≥ 35 m or ≥ 8% prediction US (American reference equation) or ≥ 6% prediction CAN (Canadian reference equation) at 6-month follow-up
Ozpelit et al. [36]	6MWD	Death at follow-upd	Patients with shorter 6MWD at baseline had a greater risk of death at follow-up^d^ in univariate analysis
Zelniker et al. [43]	6MWD	Death at 4 years	Patients with lower 6MWD at baseline had a greater risk of death at 4 years
Fritz et al. [40]	6MWD	Death at 2 years	Patients with shorter baseline 6MWD or in the lower quartiles of 6MWD had a greater risk of death at 2 years; similar findings for 6MWD at 12 weeks
Batal et al. [41]	6MWD	Death within 2 years	6MWD ≤ 250 m at baseline was independently associated with an increased risk of patients dying within 2 years relative to patients surviving ≥ 5 years
Nickel et al. [46]	6MWD	Death or lung transplant within 5 yearse	Patients with shorter baseline 6MWD had a higher risk of death or lung transplant in univariate and multivariate analysis
Benza et al. [44]	6MWD	Death at 3 years	Patients with smaller improvements in 6MWD between baseline and 12-week follow-up had an increased risk of mortality at 3 years compared with patients with ≥ 20 m increases in 6MWD Risk of mortality at 3 years decreased with each 20 m increase in 6MWD at 12-week follow-up. Overall, an increase ≥ 20 m was associated with a reduced risk of death at 3 years
Benza et al. [39]	6MWD	Death at 1 year	Patients with a baseline 6MWD < 165 m have a significantly increased risk of death at 1 year, while patients with baseline 6MWD ≥ 440 m had a significantly lower risk of death at 1 year
Humbert et al. [42]	6MWD	Death within 3 years	Patients with shorter 6MWD at baseline have a higher risk of death in individual Cox proportional hazards analysis and a multivariable Cox proportional hazards model
FC			
Snipelisky et al. [47]	NYHA	Death at follow-upf	Patients with more severe NYHA FC at baseline had an increased risk of death
Weatherald et al. [45]	NYHA	Death or lung transplant	Patients with more severe baseline NYHA FC (III/IV) had an increased risk of death or lung transplant over a median follow-up of 2.8 years (IQR: 1.1–4.6) in univariable and multivariable analyses At first follow-up (median time to first follow-up right heart catherization was 4.6 months [IQR: 3.7–7.8]), patients with more severe NYHA FC (III/IV) had an increased risk of death or lung transplant over a median follow-up of 2.8 years (IQR: 1.1–4.6) in univariable and multivariable analyses
Dufour et al. [6]	WHO	Health care resource utilization	Patients with WHO FC IV had significantly more inpatient admissions, longer average lengths of stay, and more emergency department visits than other FC subgroups
		Health care costs	Mean total health care costs for patients with PAH were higher than costs for a Centers for Medicare and Medicaid Services managed care control group and increased with more severe FC Patients in WHO FC IV have the highest costs
Tang et al. [35]	WHO	All-cause death or lung transplant	Patients with more severe WHO FC (III/IV) had an increased risk of all-cause death or lung transplant^g^
		Clinical worsening (see Table 3 for definition)	Patients with more severe WHO FC (III/IV) had increased risk of clinical worsening^g^
Ghofrani et al. [2]	WHO	Death	Patients with poor baseline WHO FC (III/IV) had significantly increased risk of death in a bivariate Cox proportional hazards model; similar findings for follow-up FC in univariate analysis Patients who improved at least one WHO FC from baseline to follow-up had a similar risk of death compared with patients whose FC did not improve, but patients who improved from WHO FC III/IV to I/II at follow-up had a reduced risk of death compared with patients who remained in WHO FC III/IV at both timepoints
		Clinical worsening (see Table 3 for definition)	Patients with poor baseline WHO FC (III/IV) or worsened FC (changing from I/II to III/IV between baseline and follow-up) had a significantly greater risk of clinical worsening in a bivariate Cox proportional hazards model; similar findings for follow-up FC in univariate analysis
Huang et al. [34]	WHO	Clinical worsening (see Table 3 for definition)	More severe baseline WHO FC (III/IV) was associated with an increased risk of clinical worsening
Ozpelit et al. [36]	NYHA	Death at follow-upd	Patients with more severe NYHA FC (III/IV) at baseline had an increased risk of death at follow-up^d^ in univariate and multivariate analysis
Ehlken et al. [53]	WHO	Health care resource utilization	Compared with patients who received medical therapy alone, patients with severe PAH who received exercise training plus medical therapy reduced their WHO FC, which was associated with less health care resource utilization
Barst et al. [49]	NYHA/WHO	Death at 3 years	Compared with those whose FC improved within 1 year of enrollment, patients whose NYHA/WHO FC worsened and those whose FC remained unchanged had an increased risk of death within 3 years This trend was stronger in a subanalysis of patients with only idiopathic/familial PAH
Tiede et al. [50]	WHO	Death or lung transplant	At follow-up (16 weeks ± 2.5 SDs; range: 4–29), patients with stable or deteriorated WHO FC had higher risk of death or lung transplant within 7 years (mean follow-up: 4.7 years) compared with patients whose FC improved in univariate Cox regression analysis
Batal et al. [41]	WHO	Death within 2 years	Baseline WHO FC IV was independently associated with an increased likelihood of patients dying within 2 years relative to patients surviving ≥ 5 years in univariate and multivariate analysis excluding initial PAH therapy
Nickel et al. [46]	WHO	Death or lung transplant within 5 yearse	Patients with more severe WHO FC (III/IV) at baseline had an increased risk of death or lung transplant in univariate analysis^e^ Patients whose FC remained IV or III or increased to III/IV during follow-up^h^ had a higher risk of lung transplant and death compared patients remaining stable at FC I/II and patients who improved from FC III/IV to I/II in multivariate analysis
Benza et al. [44]	NYHA	Death at 3 years	At baseline, patients with NYHA FC IV had an increased risk of death at 3 years compared with patients with FC III and FC II Patients with NYHA FC II had a reduced risk of death at 3 years compared with patients with NYHA FC III
Kane et al. [48]	WHO	Death within 5 yearsi	Patients with more severe WHO FC (III/IV) at baseline have an increased risk of death within 5 years,^i^ with risk increasing by 69% per class
Benza et al. [39]	NYHA/WHO	Death at 1 year	At baseline, patients with NYHA/WHO FC IV had the highest risk of death at 1-year, followed by patients with NYHA/WHO FC III Patients with modified NYHA/WHO FC-I at baseline had a significantly reduced risk of death at 1 year
Humbert et al. [42]	WHO	Death within 3 years	Patients with WHO FC I/II at baseline have a significantly lower risk of death in individual Cox proportional hazards analysis
BNP/NT-proBNP			
Frantz et al. [52]	BNP	Death at 5 years	Compared with patients with lower baseline levels (≤340 pg/mL), patients with higher baseline levels of BNP (>340 pg/mL) had a greater risk of death at 5 years Effect of change to BNP between baseline and 1-year follow-up on risk of death at 5 years: Greatest risk: patients whose BNP remains high (>340 pg/mL) Second greatest risk: patients with increasing BNP Third greatest risk: patients whose BNP decreases Lowest risk: patients whose BNP remained low (≤340 pg/mL)
Tang et al. [35]	NT-proBNP	All-cause death or lung transplant	Patients with higher NT-proBNP had an increased risk of death or lung transplant^g^ The optimal cutoff value for NT-proBNP for predicting all-cause death or lung transplant was 1,105.5 pg/mL
		Clinical worsening (see Table 3 for definition)	Patients with higher NT-proBNP had an increased risk of clinical worsening^g^
Ghofrani et al. [2]	NT-proBNP	Death	Patients with NT-proBNP higher or greater than the median (≥467 pg/mL) at baseline or increased NT-proBNP between baseline and follow-up had significantly increased risk of death in a bivariate Cox proportional hazards model; similar findings for NT-proBNP higher or greater than the median (≥268 pg/mL) at follow-up in univariate analysis
		Clinical worsening (see Table 3 for definition)	Patients with NT-proBNP higher or greater than the median (≥467 pg/mL) at baseline or increased NT-proBNP between baseline and follow-up had significantly increased risk of clinical worsening in a bivariate Cox proportional hazards model; similar findings for NT-proBNP higher or greater than the median (≥268 pg/mL) at follow-up in univariate analysis Time to first event was predicted by baseline NT-proBNP (0.91; 95% CI 0.88–0.94; *P* < 0.0001), change from baseline in NT-proBNP (0.90; 95% CI 0.85–0.95; *P* < 0.0001), and NT-proBNP at follow-up (0.91; 95% CI, 0.88–0.94; *P* < 0.0001)
Ozpelit et al. [36]	BNP	Death at follow-upd	Patients with higher BNP at baseline have an increased risk of death at follow-up^d^ in univariate and multivariate analysis
Zelniker et al. [43]	NT-proBNP	Death at 4 years	Patients with NT-proBNP > 704.5 pg/mL at baseline have a greater risk of death at 4 years
Fritz et al. [40]	BNP	Death at 2 years	Higher baseline BNP was associated with a greater risk of death over 2 years; similar findings for BNP at 12 weeks
Nickel et al. [46]	NT-proBNP	Death or lung transplant within 5 years	Patients with elevated NT-proBNP at baseline or whose NT-proBNP remained high or increased to ≥ 1,800 ng/L from baseline to follow-up^h^ had increased risk of lung transplant and death at 1, 3, and 5 years^e^ in univariate and multivariate analysis, compared with patients whose NT-proBNP was low and with patients whose NT-proBNP remained low or decreased
Kane et al. [48]	FC	Death within 5 yearsi	Patients with more severe WHO FC (III/IV) at baseline have an increased risk of death within 5 years,^i^ with risk increasing by 69% per class
Mauritz et al. [51]	NT-proBNP	Death at follow-upj	Patients with higher NT-proBNP at baseline had a greater risk of death at follow-up^j^ Patients with NT-proBNP > 1256 pg/mL at baseline have a greater risk of death at follow-up^j^ Patients with a decrease of NT-proBNP of > 15% per year at evaluation had a lower risk of death at follow-up^j^
Benza et al. [39]	BNP	Death at 1 year	At baseline, patients with BNP higher than threshold (>180 pg/mL) have a significantly higher risk of death at 1 year, while patients with BNP lower than threshold (< 50 pg/mL) have a significantly lower risk of death at 1 year
Risk groups			
Boucly et al. [54]	6MWD, FC (WHO), and BNP/NT-proBNPk	Death or lung transplant at follow-upb	Patients who achieved fewer low-risk criteria (including 6MWD > 440 m and FC I/II)^k^ at baseline or first re-evaluation^c^ have a higher risk of death or lung transplant In a subgroup analysis at first re-evaluation^c^ where BNP < 50 ng/L or NT-proBNP < 300 ng/L was added to the univariate and multivariate analyses, the number of noninvasive low-risk criteria achieved (WHO/NYHA FC I/II, 6MWD > 440 m, and BNP < 50 ng/L or NT-proBNP < 300 ng/L) significantly predicted lower risk of lung transplant or death; hemodynamic low-risk criteria were no longer significant in this model
Hoeper et al. [12]	6MWD, FC (WHO), and BNP/NT-proBNP	Death within 5 years	6MWD, FC (WHO), and BNP/NT-proBNP were the top factors determining a patient’s risk of mortality within 5 years in an analysis that also considered right atrial pressure, cardiac index, and mixed venous oxygen saturation as risk factors
Kylhammar et al. [55]	6MWD, FC (WHO), and NT-proBNP	Death within 5 yearsl	Patients in the high-risk group at baseline had the greatest risk of death within 5 years,^l^ followed by patients in the intermediate risk group, with patients in the low-risk group with the lowest risk of death at those timepoints; similar findings for risk groups at follow-up^m^ Patients with a lower proportion of variables at “low risk” at follow-up^m^ had a greater risk of death within 5 years^l^ Patients with stable intermediate risk or high risk or who worsened to intermediate risk or high risk between baseline and follow-up^m^ had a greater risk of death within 5 years^l^ compared with patients with stable low risk or who improved to low risk between baseline and follow-up^m^

*6MWD* 6-minute walk distance, *BNP* brain natriuretic peptide, *CI* confidence interval, *FC* functional class, *IQR* interquartile range, *NT-proBNP* N-terminal cleavage product of its prohormone, *NYHA* New York Heart Association, *PAH* pulmonary arterial hypertension, *Q1* first quartile, *SD* standard deviation, *US* United States, *WHO* World Health Organization

^a^Median time between the 2 6MWTs was 14.0 weeks (IQR 7.7–26.1)

^b^Over a median of 34 months (IQR 16–56)

^c^Median: 4.4 months [IQR 3.6–6.4], maximum: 1 year

^d^Followed up for mean ± SD 36.8 ± 23.6 months

^e^Median follow-up was 38 months (IQR 25–70)

^f^Mean ± SD follow-up was 4.53 ± 2.64 years

^g^Median ± SD follow-up was 41 ± 15 months (maximum: 66 months)

^h^3–12 months after initiation of PAH-targeted therapy

^i^Median follow-up of 3.2 years (IQR 1.3–5.0)

^j^Mean ± SD follow-up period of 38 ± 23 months

^k^Patient risk was assessed according to the number of low-risk criteria achieved, including the following: WHO/NYHA FC I-II, 6MWD > 440 m, right atrial pressure < 8 mm Hg, and cardiac index ≥ 2.5 min^−1^ m^−2^; risk for a subset of patients with BNP or NT-proBNP measurements available at follow-up (*n* = 630) was considered in univariate and multivariate analysis where BNP < 50 ng/L or NT-proBNP < 300 ng/L was added as an additional noninvasive low-risk criterion

^l^Follow-up was 27 (11–51) months

^m^Median time from baseline to first follow-up was 4 months (IQR 3–5)

#### 6MWD and Risk of Death

Risk of death was increased among patients with 6MWD that was shorter [2, 36], below the median (< 380 m) [2], or in lower quartiles at baseline (reference: Q1 ≤ 300 m) [37]. Specifically, risk of death at 1 year was increased among patients with shorter 6MWD [38] or 6MWD < 165 m at baseline [38, 39] and decreased among patients with 6MWD ≥ 440 m at baseline [39]. Risk of death within 2 years was increased among patients with 6MWD that was shorter or in lower quartiles at baseline [40]. Further, 6MWD ≤ 250 m at baseline was independently associated with an increased risk of patients dying within 2 years relative to patients surviving ≥ 5 years [41]. Risk of death was also increased at 3 years [42] and 4 years [43] among patients with shorter 6MWD at baseline.

At follow-up, risk of death was increased among patients with 6MWD that was shorter [2], in lower quartiles (reference: Q1 ≤ 348 m) [37], or below the median (defined as ≤ 400 m [37] or < 418 m [2]). Within 1 year, risk of death was increased among patients with 6MWD that was < 165 m [38]. Risk of death within 2 years was increased among patients with 6MWD that was shorter or in lower quartiles at 12-week assessment [40]. At 3 years, risk of death was increased among patients with smaller improvements in 6MWD between baseline and the 12-week follow-up compared with patients with ≥ 20 m increases in 6MWD [44].

#### 6MWD and Risk of Death or Lung Transplant

Risk of death or lung transplant was increased among patients with 6MWD that was shorter [34, 45] or below cutoff at baseline (≤ 342 m) [34]. Risk of death or lung transplant at 5 years was increased among patients with shorter baseline 6MWD [46].

At follow-up, risk of death or lung transplant was higher among patients with 6MWD that was shorter [45] or declined ≥ 35 m or ≥ 8% [34].

#### 6MWD and Risk of PAH-Related Death or Hospitalization

Risk of PAH-related death or hospitalization was increased among patients with 6MWD in lower quartiles at baseline (reference: Q1 ≤ 300 m) [37]. At follow-up, risk was increased among patients with 6MWD in lower quartiles or below the median (≤ 400 m) [37].

#### 6MWD and Risk of Clinical Worsening

Risk of clinical worsening increased among patients with 6MWD that was shorter [2, 34] or ≤ 342 m at baseline [34]. Risk was higher among patients with shorter [2] or decreasing 6MWD at follow-up [2, 34].

### FC

Sixteen studies evaluated the relationship between FC and death (*n* = 9), death or lung transplant (*n* = 4), experiencing a costly event indicative of clinical worsening (*n* = 3), using health care resources (*n* = 2), and incurring health care costs (*n* = 1) (Table 4).

#### FC and Risk of Death

Patients with an increased risk of death at follow-up had more severe baseline FC (III/IV) [2, 36, 47], with highest risk of death within 1 year specifically among FC IV patients [39]. Further, FC IV symptomatology at baseline was independently associated with an increased likelihood of reduced survival (dying within 2 years) in univariate and multivariate analysis excluding initial PAH therapy [41]. Risk of death within 3 years was significantly higher for patients with more severe FC (III/IV) at baseline [42, 44]. Within 5 years, risk of death was higher for FC III or IV patients at baseline and increased 69% per class [48].

Risk of death was also predicted by more severe FC (III/IV) at follow-up [2]. Patients who improved at least one FC from baseline to follow-up had a similar risk of death compared with patients whose FC did not improve, but patients who improved from FC III/IV to I/II at follow-up had a reduced risk of death compared with patients who remained in FC III/IV at both time points [2]. Risk of death at 3 years was higher for patients whose FC worsened or remained unchanged compared with those whose FC improved within 1 year of enrollment [49].

#### FC and Risk of Death or Lung Transplant

For the combined endpoint of risk of death or lung transplant, risk was higher among patients with more severe FC (III/IV) at baseline in studies examining FC [35, 45]. Risk of death or lung transplant within 5 years was higher for patients with more severe FC (III/IV) at baseline [46].

Risk of death or lung transplant was also predicted by more severe FC (III/IV) [45] at follow-up and changes in FC between baseline and follow-up. Risk of death or lung transplant within 5 years was increased for patients whose FC remained III or IV or increased to III/IV during follow-up compared with patients who remained at FC I/II and patients who improved from FC III/IV to I/II [46]. Within 7 years, risk of death or lung transplant similarly increased for patients with stable or deteriorated FC compared with patients whose FC improved [50].

#### FC and Risk of Clinical Worsening

Risk of clinical worsening was increased among patients with more severe FC (III/IV) at baseline [2, 34, 35]. More severe FC (III/IV) at follow-up or FC that worsened (from I/II to III/IV) between baseline and follow-up also predicted clinical worsening [2].

### BNP/NT-proBNP

Nine studies evaluated the relationship between BNP/NT-proBNP and risk of death (*n* = 7), experiencing a costly event indicative of clinical worsening (*n* = 2), and death or lung transplant (*n* = 2) (Table 4). The frequency of use of BNP (four studies) and NT-proBNP (five studies) was similar in the literature, with no clear temporal differences by year of publication or study start date.

#### BNP/NT-proBNP and Risk of Death

Risk of death was increased for patients with higher BNP [36] or NT-proBNP at baseline [2, 51] or NT-proBNP above the median (≥ 467 pg/mL) [2] or cutoff (>1256 pg/mL) at baseline [51]. Risk of death at 1 year was increased for patients with BNP higher than the threshold (> 180 pg/mL) at baseline [39]. Risk of death at 2 years was increased among patients with higher BNP at baseline [40]. At 4 years, risk of death was increased among patients with NT-proBNP > 704.5 pg/mL at baseline [43]. Increased risk of death at 5 years was associated with baseline levels of BNP > 340 pg/mL [52].

Risk of death also increased for patients whose NT-proBNP was higher [2], greater than the median (≥ 268 pg/mL) [2], or increased between baseline and follow-up [2, 51]. Risk of death at 2 years, in particular, was increased among patients with elevated BNP at the week 12 assessment [40]. Risk of death at 5 years was highest among patients whose BNP remained high (> 340 pg/mL), followed by patients with increased BNP at 1 year of follow-up; patients whose BNP decreased or remained low (≤ 340 pg/mL) at 1 year of follow-up had the lowest risk [52].

#### NT-proBNP and Risk of Death or Lung Transplant

Risk of death or lung transplant was increased for patients with elevated NT-proBNP at baseline [35, 46]. An optimal cutoff of NT-proBNP > 1105.5 pg/mL at baseline predicted risk of all-cause death or lung transplant [35]. Risk of death at 1, 3, and 5 years was increased among patients whose NT-proBNP remained high or increased to ≥ 1800 ng/L at follow-up [46].

#### NT-proBNP and Risk of Clinical Worsening

Risk of clinical worsening was increased among patients with NT-proBNP that was elevated [2, 35] or greater than the median (≥ 467 pg/mL) at baseline [2]. Risk of clinical worsening was also increased among patients with elevated or increased NT-proBNP at follow-up [2].

#### FC and Economic Outcomes

Mean total health care costs for patients with PAH were higher than costs for a Centers for Medicare and Medicaid Services managed care control group and increased with more severe baseline FC, with patients in FC IV having the highest costs [6]. Health care resource utilization, including inpatient admissions, longer average lengths of stay, and emergency department visits, was also greater for patients with FC IV than other FC subgroups [6]. In another study, patients with severe PAH who received medical therapy alone were more likely to have more severe FC (III/IV), which was associated with greater health care resource utilization compared with patients who received exercise training plus medical therapy [53].

#### 6MWD, FC, and BNP/NT-proBNP Risk Groups

Three studies evaluated the relationship between risk group and death within 5 years (*n* = 2) and death or lung transplant (*n* = 1) (Table 4). The 2015 European Society of Cardiology (ESC)/European Respiratory Society (ERS) guidelines were used to stratify patients as low, intermediate, or high risk [12, 54, 55]. All three studies considered FC and 6MWD when determining risk [12, 54, 55] (Table 4). Two considered BNP/NT-proBNP in the primary determination of risk [12, 55], while one study considered the additive value of BNP < 50 ng L^−1^ or NT-proBNP < 300 ng L^−1^ low-risk criteria [54]. Additional factors considered in determining risk included right atrial pressure, cardiac index, mixed venous oxygen saturation [12, 54, 55], right atrial area, and pericardial effusion [55]. Risk was determined by a mean score calculated using the sum of the grades assigned to each risk factor from 1 (low risk) to 3 (high risk) and dividing by the total number of risk factors [12, 55] and by the number of low-risk criteria present at baseline and re-evaluation (FC I-II, 6MWD > 440 m, right atrial pressure < 8 mm Hg and cardiac index ≥ 2.5 min^−1^ m^−2^) [54].

#### Risk Groups and Risk of Death

Risk of death within 5 years was increased among patients with a higher proportion of “high-risk” variables at both baseline and follow-up, determined with the inclusion of 6MWD, FC, and NT-proBNP [55]. 6MWD followed by FC and BNP/NT-proBNP most strongly correlated with a patient’s risk of death within 5 years [12].

#### Risk Groups and Risk of Death or Lung Transplant

At baseline, all four low-risk criteria significantly predicted risk of death or lung transplant in univariable analysis; 6MWD > 440 m was the only low-risk criterion remaining significant in multivariable analysis [54]. At first re-evaluation, all four low-risk criteria significantly predicted risk of death or lung transplant in univariate and multivariable analysis [54]. Outcomes were similar for patients who had an increase in the number of low-risk criteria achieved between baseline and first re-evaluation (< 3 to having 3–4) and those who had 3–4 low-risk variables at both time points. Patients with less than three low-risk criteria at both baseline and first revaluation had the greatest risk of death or lung transplant; for patients with zero low-risk variables at follow-up, transplant-free survival was worse for those with two high-risk variables than for those with one. In the subgroup of patients who had all three noninvasive measurements at follow-up, risk of death or lung transplant was significantly lower for patients who achieved one or more low-risk criteria (FC I-II, 6MWD > 440 m, BNP < 50 ng L^−1^); in this multivariable model, hemodynamic low-risk criteria (right atrial pressure < 8 mm Hg and cardiac index ≥ 2.5 min^−1^ m^−2^) were no longer significant predictors of transplant-free survival [54].

## Discussion

This review provides support for 6MWD, FC, and BNP/NT-proBNP as correlates of risk of long-term health outcomes (e.g., mortality and clinical worsening), costly events (e.g., lung transplants or hospitalization), and economic outcomes (e.g., costs and resource utilization) in PAH. Relative to patients with longer or increased 6MWD, patients with shorter or decreased 6MWD have a higher risk of death and experiencing costly events indicative of clinical worsening, such as hospitalization or lung transplant. Compared to patients with more favorable (I or II) or improved FC, patients with poorer (III or IV) or declined FC consume greater health care resources, incur higher health care costs, and are at an increased risk of death and clinical worsening. Patients with elevated or increased BNP/NT-proBNP have a higher risk of death and clinical worsening relative to patients with lower or decreased BNP/NT-proBNP. In addition, patients classified into more severe risk groups, determined by multiple noninvasive endpoints (e.g., 6MWD, FC, and BNP/NT-proBNP), have an increased risk of death and lung transplant.

These findings are important considering the current shift from using these noninvasive measures as primary and secondary endpoints in PAH clinical trials to evaluating mortality and clinical worsening in clinical trials. A related review has also concluded that 6MWD and FC are clinically meaningful trial endpoints associated with outcomes in patients with PAH and CTEPH [16]. While mortality and clinical worsening are robust and valuable long-term endpoints in clinical trials, 6MWD, FC, and BNP/NT-proBNP remain informative prognostic indicators that can be used in the clinic to assess and reduce risk of death or costly events for patients with PAH [15]. Whereas multiple definitions are used for clinical worsening across trials, 6MWD, FC, and BNP/NT-proBNP are universally defined and recognized, enabling comparisons of treatment efficacy across trials using these endpoints. 6MWD and FC are already being included in the composite endpoint of clinical worsening in many trials and in risk calculators such as REVEAL 2.0 [13]. BNP/NT-proBNP is also included in risk calculators [13] and is a prominent biomarker used in clinical management [56, 57] as well as part of the multiparametric risk assessment approach outlined in guidelines [58]. These noninvasive endpoints are simpler and more inexpensive to assess relative to event-based endpoints [14]. For instance, morbidity and mortality event trials require a large number of subjects in order to demonstrate effects even as early as 1 year [59, 60]. Looking to the future, consideration should be given to including 6MWD, FC, and BNP/NT-proBNP in the assessment of time to clinical improvement, a new endpoint proposed at the recent 6th World Symposium on Pulmonary Hypertension [61].

Conclusions from the present review should be considered with caution because no pooled analysis was performed to evaluate the observed relationships between the noninvasive endpoints and long-term outcomes for statistical significance. Previous meta-analyses and pooled analyses for 6MWD have found no correlation between 6MWD and mortality [21] and clinical events [22] or only modest validity for 6MWD as a surrogate endpoint [18]. Given that the majority of studies evaluating 6MWD included in this review were published after these studies were conducted (10 of 13 studies), an updated analysis is needed in order to confirm our observations. In addition, the paucity of literature evaluating health care resource utilization and costs in PAH limits the ability to draw conclusions on the predictive nature of noninvasive endpoints in terms of economic outcomes. However, given what is known about the relationships between noninvasive endpoints and events, such as hospitalization and lung transplant, the economic impact may be extrapolated using existing data on the costs of those events. Further, our review did not consider the predictive value of additional variables that are considered in risk assessments such as cardiopulmonary exercise testing, imaging (e.g., echocardiogram, cardiac magnetic resonance imaging), hemodynamics, and right heart catherization [13, 56]. While these variables are important, our review focused on the noninvasive endpoints most commonly used in clinical trials and risk assessments that are most efficient and inexpensive to assess.

## Conclusions

Mortality and clinical worsening will continue to be valuable endpoints in assessing treatment efficacy and safety in PAH; however, these endpoints require lengthy follow-up and cannot be applied in clinical settings where risk reduction is the goal of treatment. Further, differing definitions used for clinical worsening across trials limit the ability of stakeholders to compare treatment efficacy. 6MWD, FC, and BNP/NT-proBNP are universally defined, low-cost, efficient, noninvasive endpoints that correlate with long-term health and economic outcomes in patients with PAH. Collectively, they are important components of risk assessments and will remain beneficial in the clinic to guide treatment decisions. Future research should meta-analytically examine the relationships between these noninvasive endpoints and long-term outcomes for statistical significance. Additional studies are needed examining the relationship between 6MWD, FC, and BNP/NT-proBNP and economic outcomes and the potential utility of 6MWD, FC, and BNP/NT-proBNP as a composite endpoint assessing risk.
